# Statistical Optimization of Media Components for Production of Fibrinolytic Alkaline Metalloproteases from *Xenorhabdus indica* KB-3

**DOI:** 10.1155/2014/293434

**Published:** 2014-04-23

**Authors:** Kumar Pranaw, Surender Singh, Debjani Dutta, Surabhi Chaudhuri, Sudershan Ganguly, Lata Nain

**Affiliations:** ^1^Division of Microbiology, Indian Agricultural Research Institute, New Delhi 110012, India; ^2^Department of Biotechnology, National Institute of Technology, Durgapur 713209, India; ^3^Division of Nematology, Indian Agricultural Research Institute, New Delhi 110012, India

## Abstract

*Xenorhabdus indica* KB-3, a well-known protease producer, was isolated from its entomopathogenic nematode symbiont *Steinernema thermophilum*. Since medium constituents are critical to the protease production, the chemical components of the selected medium (soya casein digest broth) were optimized by rotatable central composite design (RCCD) using response surface methodology (RSM). The effects of all five chemical components (considered as independent variables), namely tryptone, soya peptone, dextrose, NaCl, and dipotassium phosphate, on protease production (dependent variable) were studied, and it was found that tryptone and dextrose had maximum influence on protease production. The protease production was increased significantly by 66.31% under optimal medium conditions (tryptone—5.71, soya peptone—4.9, dextrose—1.45, NaCl—6.08, and dipotassium phosphate—0.47 in g/L). To best of knowledge, there are no reports on optimization of medium component for protease production by *X. indica* KB-3 using RSM and their application in fibrinolysis. This study will be useful for industrial processes for production of protease enzyme from *X. indica* KB-3 for its application in the field of agriculture and medicine.

## 1. Introduction

*Xenorhabdus* is a unique genus of bacteria, which is symbiotically associated with entomopathogenic nematode of genus* Steinernema*.* Xenorhabdus* spp. are known to produce a plethora of secondary metabolite-like antibiotics (xenorhabdins, xenorxides, xenocoumacins, indole derivatives, nematophin, and xenorhabdicin), toxins (both endo and exo), and extracellular enzymes, namely, lipases, esterases, chitinase, phospholipases, and proteases. These metabolites not only have diverse chemical structures, but also have a wide range of bioactivities with medicinal and agricultural interests. Proteolytic enzymes are recognized by their catalytic type and are classified as aspartic, cysteine, metallo, serine, threonine, and several others that are yet to be classified. Most proteolytic enzymes are classified as metalloproteases. Several researchers have documented the secretion of metalloproteases by* Xenorhabdus* strains previously [1–3]. The* Xenorhabdus indica *KB-3 isolated in the present study produces serralysin type of proteases [4] like few other* Xenorhabdus* [1, 2, 5, 6]. Serralysin differs from other metalloproteases through their mode of action. Serralysin specially cleaves the peptide bonds, which are associated with hydrophobic residues [7]. Serratiopeptidases have potent anti-inflammatory properties and are particularly useful in posttraumatic swelling, fibrocystic breast disease, and bronchitis. Fibrin is the primary protein constituent of blood clots, which are formed by thrombin. Fibrinolytic activity of serratiopeptidases makes them useful candidates to digest blood clots [8–10]. Due to these special characteristics, this miracle enzyme has attracted medical interest and its potency as a clinical agent to fight against different diseases is already proved. Isolation of these kinds of metalloproteases from new sources and their mass production are much desired. Thirty to forty percent of the manufacture cost of enzyme production industrially is estimated to be the cost of growth medium [11]. For mass production, designing an appropriate fermentation medium is of critical importance in optimizing the product yield. The one-variable-at-a-time is the most frequently used conventional experimental approach, but this strategy not only is time consuming but also requires a large number of experiments because the effect of each factor on enzyme production needs to be investigated individually, and their interaction effect on production process cannot be quantified exactly [12]. On the contrary, statistical optimization of media components by response surface methodology (RSM) includes central composite design for different variables. RSM helps in evaluating the significant factors, model designing to study interaction between different variables and to select the optimum value of variables for maximum desirable response [13, 14]. Therefore, in this study, response surface methodology (RSM) was used to optimize the media components for maximum production of metalloproteases from* X. indica* KB-3 and its application in fibrinolysis.

## 2. Materials and Methods

### 2.1. Symbiotic Bacterial Strain and Culture Conditions

*X. indica* strain KB-3, a nematode symbiont, was isolated from* Steinernema thermophilum* obtained from the soil of Kerala, India. The strain had been identified to be* X. indica* according to its morphological and molecular characteristics [4].* X. indica* KB-3 was maintained on nutrient agar (NA) slants supplemented with 0.04% (w/v) triphenyltetrazolium chloride and 0.025% (w/v) bromothymol blue and subcultured monthly [15]. Seed culture for protease production was prepared by inoculating a single colony of phase I* X. indica* KB-3 into 50 mL fresh soya casein digest medium [2]. The inoculated flasks were incubated at 28°C in an incubator shaker (150 rpm) for 48 h.

### 2.2. Screening of the Optimal Nutrient Medium for Protease Production

For optimum production of protease enzyme, initially, five different media combinations were screened to find a suitable nutrient medium for protease enzyme production, which are as follows:

1. nutrient broth (peptone—5 g, beef extract—3 g, and NaCl—4 g per liter) supplemented with skim milk (1%) and gelatin (1%) separately,
2. nutrient broth half strength (peptone—2.5 g, beef extract—1.5 g, and NaCl—2 g per liter) supplemented with skim milk (1%) and gelatin (1%) separately,
3. soya casein digest medium (tryptone—17 g, NaCl—5 g, soya peptone—3 g, dipotassium phosphate—2.5 g, and dextrose—2.5 g per liter) (all chemicals were purchased from HiMedia Laboratories Pvt. Ltd., India).

The media were inoculated with 2% (v/v) seed culture into 50 mL of respective medium and incubated at 28°C, 150 ×g rpm for 24 h. Samples were withdrawn at intervals of 3 h up to 24 h and centrifuged at 10,000 rpm for 5 min. The cell pellet was discarded, and the supernatant was preserved at 4°C for enzyme analysis.

### 2.3. Optimization of Physiochemical Variables for Maximum Production of Protease Enzyme

Tryptone, soya peptone, dextrose, NaCl, and dipotassium phosphate were considered as chemical components while pH, temperature, incubation time, and inoculum percentage were considered as physical parameters for protease production from* X. indica* KB-3. Before optimization, the important factors were screened by Plackett-Burman (PB) factorial design. After screening, optimization was done by rotatable central composite design (RCCD) using response surface methodology (RSM) by experimental design module of Design Expert-7 (Stat-Ease Inc., Minneapolis, USA). Optimization of the chemical components and physical parameters was carried out separately assuming no interaction took place between these factors. Initially physical parameters were optimized using RSM separately (data not shown) followed by chemical optimization under optimal physical conditions (pH—7.7, temperature—28.0°C, incubation time—18 h, and inoculum percentage—3%).

### 2.4. Selection of Significant Chemical Variables by the Plackett-Burman (PB) Design

In preliminary studies, “one-factor-at-a-time” approach was used to evaluate various factors for their suitability to sustain good production of protease by* X. indica* KB-3. To determine the significant chemical factors for protease production, a total of five chemical components at three levels (i.e., −1, 0, and +1) were involved in the determination of chemical components: (1) tryptone, (2) soya peptone, (3) dextrose, (4) NaCl, and (5) dipotassium phosphate. Tables 1(a) and 1(b) list the media components, codes, and levels of the different variables of the experimental design. The principal effects of each variable on the protease activity were estimated as the difference between the averages of measurements obtained at both the higher and the lower level. The significance of each variable was determined via Student's *t*-test.

### 2.5. Optimization of Chemical Variables by RSM

All five chemical components, (*A*) tryptone, (*B*) soya peptone, (*C*) dextrose, (*D*) NaCl, and (*E*) dipotassium phosphate, were selected to find the optimum chemical components for protease enzyme from* X. indica* KB-3 using RCCD. The ranges and levels of the variables taken for RSM are listed in Table 2(a). According to RCCD, the total number of experimental combinations is 2^*k*^ + 2*k* + *n*_*o*_, where *k* is the number of independent variables and *n*_*o*_ is the number of repetitions of the experiments at the centre point. A total of 50 sets of experiments including eight center points were conducted along with different combination of chemical components. Each numeric factor is varied over 5 levels (−2.378, −1, 0, +1, and +2.378), that is, plus and minus alpha (axial point), plus and minus one (factorial points), and zero (center point). The response values (*Y* = protease activity) in each trial were the average of the triplicates. The full experimental plan along with coded variables is mentioned in Table 2(b).

### 2.6. Statistical Analysis and Validation of Experimental Modeling

The data obtained from RSM were subjected to analysis of variance (ANOVA) for analysis of regression coefficient, prediction equations, and case statistics. The experimental results of RSM were fitted using the following second order polynomial equation:

(1) $$\begin{array}{c} Y=\beta o+\underset{i}{\sum }\beta iXi+\underset{ii}{\sum }\beta iiXi^{2}+\underset{ij}{\sum }\beta ijXiXj. \end{array}$$

In this polynomial equation, *Y* is the predicted response, *Xi* and *Xj* are independent variables, *βo* is the intercept term, *βi* is the linear coefficient, *βi* *i* is the quadratic coefficient, and *βi* *j* is the interaction coefficient. The statistical model was validated with respect to all variables within the design space. A random set of 5 experimental optimized combinations were used to study the protease production under submerged fermentation.

### 2.7. Enzyme Assay

The culture broth centrifuged at 10,000 rpm for 5 min was used as enzyme source. Protease activity was measured by incubating 150 *μ*L enzyme solution and 250 *μ*L of 2% w/v azocasein (Megazyme) in water bath at 30°C for 30 min [16]. After incubation 1.2 mL of 10% trichloroacetic acid was added to stop the reaction and the mixture was allowed to stand for 15 min followed by centrifugation at 10,000 rpm for 5 min to remove any undigested azocasein. NaOH (1.4 mL) was added to final reaction supernatant and OD was determined using spectrophotometer at 440 nm. The one unit of enzyme was defined as the amount of enzyme required to produce an absorbance change of 0.01 in 1 cm cuvette under the standard assay conditions.

### 2.8. Purification and Characterization of Protease Enzyme

Extracellular protease enzyme was extracted from cell suspension grown under optimized soya casein digest medium after 18 h of incubation by centrifugation at 10,000 rpm. Purification and characterization of protease enzyme were carried out as described previously [4].

### 2.9. Fibrinolytic and Fibrinogenolytic Assays

Fibrin degradation analysis was performed by modified method of Datta et al. (1995) and Mahajan et al. (2012) [17, 18]. The fibrin gel plate of 1 mm thickness was made by cross-linking of fibrinogen in presence of thrombin. Both fibrinogen and thrombin solutions were prepared in 20 mM Tris-HCl (pH 8.2) containing 0.15 M NaCl. Five microliters of human fibrinogen solution (10 mg/mL) was added to 0.5 mL of human thrombin (50 unit/mL) and allowed to stand for 1 h at room temperature. Then 10 *μ*L of the protease enzyme was inoculated onto the plate using agar well assay method. The plate was incubated for 5 h at 37°C and the presence of the clear hydrolytic zone was measured. A clear zone of fibrin hydrolysis confirms the presence and the potency of the fibrinolytic activity. Plasmin from human plasma (Sigma-Aldrich) was used as positive control.

## 3. Results

### 3.1. Screening of the Optimal Nutrient Medium for Protease Enzyme Production

Among different growth media, the highest proteolytic activity (955.68 U/mL) was observed in soya casein digest broth after 18 h, and 832 U/mL activity was observed with skim milk supplemented nutrient broth. Therefore, soya casein digest broth was selected for further optimization study using statistical approaches. Physical parameters were optimized first (optimized physical factors: pH—7.7, temperature—28.0°C, incubation time—18 h, and inoculum percentage—3%) followed by optimization of different chemical components of soya casein digest medium using COVT method, Plackett-Burman design, and response surface methodology (RSM).

### 3.2. Optimization of Chemical Factors by Change One Variable Per Time (COVT) Method

**Figure 1:**
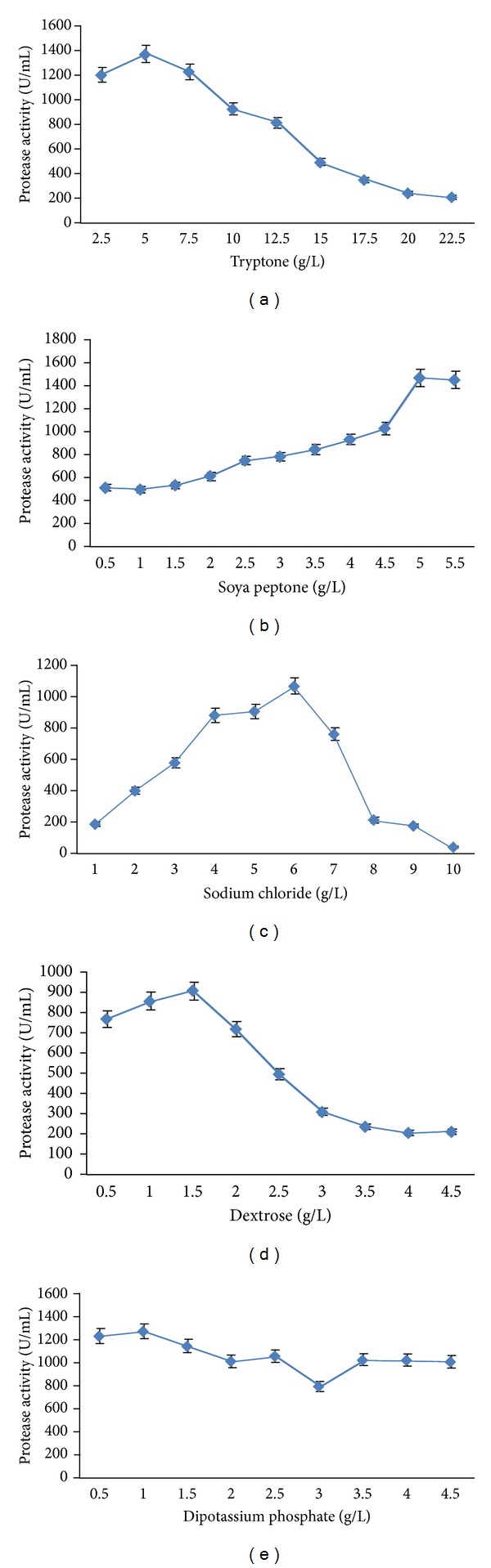
Optimization of chemical components under COVT approach for maximum production of protease by* X. indica *strain KB-3.

Standard chemical composition of soya casein digest broth in g/L was tryptone—17.0, soya peptone—3.0, sodium chloride—5.0, dextrose—2.5, and dipotassium phosphate—2.5. The effect of the five factors, namely, tryptone, soya peptone, sodium chloride, dextrose, and dipotassium phosphate, was studied on protease production using COVT method. Protease production was observed over a broad tryptone range (2.5 to 22.5 g/L). The optimum tryptone concentration for protease production was determined to be 5.0 g/L. Protease production increased consistently with the increase in tryptone concentration from 2.5 to 5.0 g/L and decreased with further increase in tryptone concentration (Figure 1(a)).The optimum concentration of soya peptone for protease production by* X. indica* strain KB-3 was estimated to be 5 g/L, producing 1453 U/mL within 18 hrs (Figure 1(b)).

The effect of salinity on protease production in* X. indica* was studied in terms of NaCl concentration. The NaCl concentration was varied from 1 to 10 g/L and maximum protease production was observed at 6 g/L NaCl concentration (Figure 1(c)). The effect of dextrose and dipotassium phosphate was also studied on protease production. Slight improvement in protease production was observed when dextrose concentration increased from 0.5 to 1.5 g/L, and beyond this concentration, the activity gradually decreased. Optimum concentration of dextrose was found to be 1.5 g/L (Figure 1(d)). The optimum concentration of dipotassium phosphate was found to be 1.0 g/L (Figure 1(e)).

### 3.3. Selection of Significant Chemical Variables by the Plackett-Burman (PB) Design

A total of five variables were analyzed for their effects on protease production using Plackett-Burman design. The PB design matrix selected for the screening of chemical components for protease production and their respective response are shown in Table 1(b). Estimated effect and analysis of variables for protease production from PB design experiment are shown in Table 3. In this study, only three factors, namely, soya peptone (*B*), dextrose (*C*), and NaCl (*D*), having low *P* value (*P* value < 0.05) were identified to be the significant variables for protease production, but tryptone (*A*) and dipotassium phosphate (*E*) were identified as insignificant variables because of high *P* value. Tryptone is the main protein source of the medium, and during COVT experiments, it was found that the tryptone is one of the important factors and had major influence on protease activity, whereas potassium is a known inducer for alkaline proteases [19]. Therefore, all of the above five factors were considered for further optimization studies. A large studentized effect, either positive or negative, indicates a large impact on response. All chemical components except dipotassium phosphate had a positive effect on the protease production. The acceptability of the model was calculated and the variables showing statistically significant effects were screened via Student's *t*-test for ANOVA (Table 3). The Model *F*-value of 19.00 implies the model is significant. There is only a 0.13% chance that a Model *F*-value could occur due to noise. The predicted *R*-squared of 0.7624 is in reasonable agreement with the adjusted *R*-squared of 0.8911. An adequate precision measures the signal to noise ratio. A ratio greater than 4 is desirable. In this study, model ratio of 13.562 indicated an adequate signal.

### 3.4. Optimization of Different Chemical Variables Using RSM

RSM was used to evaluate the relationship between different independent variables and their interactive effects on protease production by* X. indica* KB-3. All five chemical variables which showed effect on protease production in PB design, namely, tryptone (*A*), soya peptone (*B*), dextrose (*C*), NaCl (*D*), and dipotassium phosphate (*E*), were examined through RSM following RCCD matrix. The experimental data along with the actual and predicted yield of protease obtained is given in Table 2(b). The results obtained after RCCD were subjected to analysis of variance (ANOVA) and fitted with the polynomial equation (1).

Following quadratic model of response equation in terms of coded variable and actual variables was obtained as follows:

(2) Y=+1569.56+19.81∗A−2.04∗B−18.00∗C+7.94∗D−8.59∗E−59.39∗A∗B+27.83∗A∗C−12.80∗A∗E−23.25∗B∗C−25.11∗C∗D+33.59∗C∗E−29.98∗D∗E−42.10∗A2−55.18∗B2−48.37∗C2−73.92∗D2−78.69∗E2,Protease=−11425.24+395.95∗Tryptone+2639.51∗Soya  Peptone+902.98∗Dextrose+1090.20∗NaCl+2928.19∗Dipotassium  Phosphate−59.39∗Tryptone∗Soya  Peptone+27.83  ∗Tryptone∗Dextrose−25.59∗Tryptone∗Dipotassium  Phosphate−93.01∗Soya  Peptone∗Dextrose−50.22∗Dextrose∗NaCl+268.73∗Dextrose∗Dipotassium  Phosphate−119.94∗NaCl∗Dipotassium  Phosphate−10.52∗Tryptone2−220.71∗Soya  Peptone2−193.49∗Dextrose2−73.92∗NaCl2−1259.02∗Dipotassium  Phosphate2,

where *Y* is the response (protease activity in U/mL) and *A*, *B*, *C*, *D*, and *E* are the coded values of the independent variables, namely, tryptone, soya peptone, dextrose, NaCl, and dipotassium phosphate, respectively.

The ANOVA showed suitability of the model for protease production. The quadratic type model was produced, and all the effects were considered significant (*P* < 0.05). The linear terms of *A*, *C*, and *E* were significant at *P* < 0.05 level. The result showed that linear effect of *B* and *D* as well as the interactive effects of *AD*, *BD*, and *BE* was not significant, and therefore these terms were removed from final model equation. After excluding insignificant model terms *B* and *D*, models become nonhierarchical. To avoid nonhierarchy, only interactive terms *AD*, *BD*, and *BE* were excluded and parameters were recalculated (Table 4). Among the linear terms, the main effects of tryptone and dextrose on protease enzyme production were most significant as evident from their respective *P* values (*P*_*A*_ ≤ 0.0001 and *P*_*C*_ = 0.0002). The aptness of the model was checked by the coefficient of determination (*R* ^2^), which was calculated to be 0.9747, indicating that 97.47% of the variability in the responses could be explained by the model. The computed *F*-value of 0.83 suggests insignificance of the lack of fit. The model was found to be highly significant and sufficient to exemplify the actual relationship between the response and the significant variables as indicated by the small model *P* value (*P* < 0.05), large lack-of-fit sum of square value (19313.66). The predicted coefficient of determination (predicted *R* ^2^ = 0.9384) is in reasonable agreement with the adjusted coefficient of determination (adjusted *R* ^2^ = 0.9612). Significance of fifteen model terms (*A*, *C*, *E*, *AB*, *AC*, *AE*, *BC*, *CD*, *CE*, *DE*, *A* ^2^, *B* ^2^, *C* ^2^, *D* ^2^, and *E* ^2^) and an adequate precision of 27.29 acknowledged low signal to noise ratio (a ratio greater than 4 is desirable) (Table 4).

### 3.5. Response Surface Analysis for Influence of Variables on Protease Production

**Figure 2:**
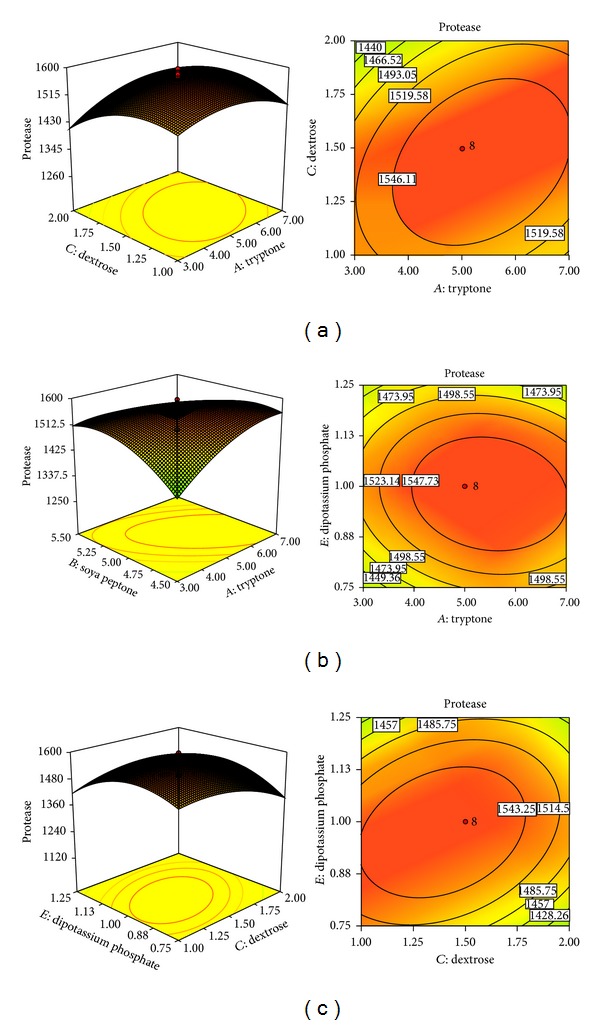
Response surface plot and respective contour plot. (a) The combined effect of tryptone and dextrose, (b) the combined effect of tryptone and dipotassium phosphate, and (c) the combined effect of dextrose and dipotassium phosphate on protease production of* X. indica* KB-3.

To analyze the effects of the independent variables and interactive effects of each independent variable for maximum protease production, 3D response surface curves and the 2D contour plots were drawn against two experimental variables while the other variables were maintained constant at its central level (Figure 2).

Figure 2(a) showed the effect of tryptone and dextrose on protease production while the other three variables were fixed at their middle level (soya peptone—5, NaCl—6 and, dipotassium phosphate—1 in g/L). Increasing tryptone concentration from 3.0 to 7.0 g/L significantly increased the protease activity from 1350 to 1596 U/mL at a low dextrose concentration (1.5 g/L), but thereafter no significant increase in protease activity was observed. Results also showed that when the initial dextrose was beyond level +*α*, the protease activity decreased. The 3D plot and its respective contour plot expedited the identification of the optimal levels of tryptone and dextrose. The optimal concentration of tryptone was around 5.71 g/L and dextrose was around 1.45 g/L.

Figures 2(b) and 2(c) showed the interaction of variables dextrose, tryptone, and dipotassium phosphate and their interaction on protease production. When tryptone concentration was near neutral value, the increase of dipotassium phosphate concentration from 0.75 to 1.13 g/L resulted in higher protease production. The optimal concentration of dipotassium phosphate was observed near 0.97 g/L. There was significant interaction between dextrose and dipotassium phosphate (Figure 2(c)).

**Figure 3:**
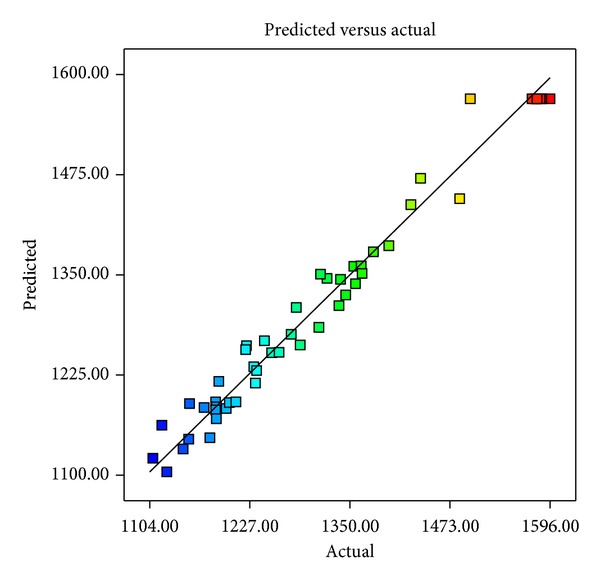
Residual diagnostics of contour surface of the quadratic model by predicted versus actual protease production of* X. indica* KB-3.

Optimization of enzyme production was carried out numerically by using Design Expert software, version 8.0.7.1, to evaluate the optimum values for each variable from the model. In optimization process, the goal for each variable was to select a range which could provide the highest protease activity. On the basis of experimental design and developed model, the experiment was carried out under optimal conditions for maximum protease production by* X. indica* KB-3. The predicted protease activity was 1575.11 U/mL under the optimum conditions, that is, tryptone—5.71, soya peptone—4.9, dextrose—1.45, NaCl—6.08, and dipotassium phosphate—0.47 in g/L. The model was validated by comparing the observed and predicted value from model equation (1). Points above or below the diagonal line showed area of over-and underprediction for protease activity (Figure 3).

For validation of overall model and optimized condition, experiments were carried out under predicted optimal conditions. The experimental protease activity 1589.42 U/mL under optimized condition was in close agreement with the predicated protease activity. Therefore, the model developed was reliable for predicting the optimal conditions for variables influencing the protease production by* X. indica* KB-3.

### 3.6. Fibrinolytic Activity of Purified Protease

**Figure 4:**
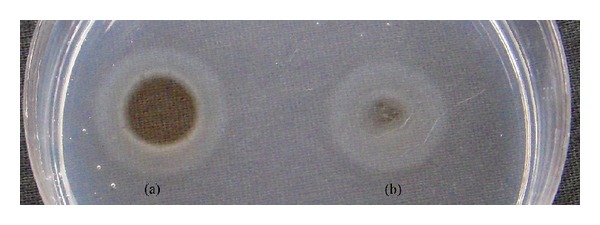
Fibrinolytic activity of (a) purified and (b) crude protease in fibrin degradation assay.

Fibrin plate method for fibrinolytic activity was tested and found positive for purified protease enzyme. After incubation at 37°C, plates of fibrin inoculated with protease showed relatively bigger zone (15 mm) of hydrolysis than that of plasmin (Figure 4), which confirms fibrinolytic activity of purified protease and its serralysin kind of nature.

## 4. Discussion

*X. indica* is a potent producer of proteolytic enzymes and medium composition can significantly affect their production. This is the first ever optimization study on alkaline metalloproteases produced from* Xenorhabdus* sp. This study was concentrated on the optimization of the medium composition for the protease production from* X. indica *KB-3 through central composite design falling under response surface methodology (RSM) by experimental design module of Design Expert-7 (Stat-Ease Inc., Minneapolis, USA). The use of RSM empowers a better understanding of the possible interactions between all chemical component variables. The model developed in the present study supports the analysis of the influence of the chemical components on protease activity of* X. indica* KB-3. The CCD experiment was very helpful in deciding the optimal concentrations of individual chemical components and determining the interaction effects among the factors. Significant interaction between tryptone and soya peptone (*AB*), tryptone and dextrose (*AC*), tryptone and dipotassium phosphate (*AE*), soya peptone and dextrose (*BC*), dextrose and NaCl (*CD*), dextrose and dipotassium phosphate (*CE*), and NaCl and dipotassium phosphate (*DE*) revealed their importance in the production medium for maximum production of protease enzyme. The regression analysis showed that the interaction of different chemical variables opted in this study was significant. The model was validated by comparing the observed and predicted values at the optimal value and experiments were carried out under optimized condition. After optimization, an overall 66.31% increase in protease activity was achieved, which confirms the reliability of model for predicting the optimized condition.

Further purification and characterization of protease were carried out and approximately 8-fold purification was achieved. Characterization of protease, based on different pH and temperature, showed optimum protease activity at 34°C at pH 8.2. Different protease inhibitor study and MALDI-TOF/TOF analysis revealed its resemblance with serralysin type alkaline metalloproteases from* Xenorhabdus nematophila* (NCBI accession number: gi/300724813) [4].

Fibrinolytic activity confirms the serralysin nature of purified alkaline metalloproteases of* X. indica*. Area of zone of hydrolysis on fibrin plate was around 15 mm, which is almost equal to that of plasmin (16.5 mm). Therefore, it can be assumed that protease of the present study has stronger fibrinolytic activity like plasmin. The size of the clear hydrolytic zone did not change in the presence of plasminogen, suggesting that protease was a plasmin-like protease, which could directly degrade fibrin but not plasminogen activators such as urokinase (UK), streptokinase (SK), and tissue plasminogen activator (tPA). Therefore, secondary effects such as platelet activation related to plasmin formation could be avoided [20]. This is a specific advantage of serralysin over clinically used plasminogen activators. This specific metalloprotease from* X. indica* shares plasmin-like protease character, where it can be effectively used as new resource for thrombolytic agents and can be used to develop therapeutic agents for the treatment of thrombosis. The formulation may also find several biotechnological applications in ointments for topical application to control the skin inflammations.

## 5. Conclusions

To the best of our knowledge, this is the first report on optimization of medium components for protease production by* X. indica* KB-3 using RSM and their use in fibrinolysis. The optimized medium resulted in 66.31% increase of protease production and the model is found efficient, simple, less time consuming, and importantly significant. This work will be useful for industrial processes for production of protease enzyme from* X. indica* KB-3 and it also expedites its use in the field of medicine.

## Figures and Tables

**Table:** (a)

Variable	Unit	Code	Experimental value		
			Lower (−1)	Center (0)	Higher (+1)
Tryptone	g/L	*A*	3.0	5.0	7.0
Soya peptone	g/L	*B*	4.5	5.0	5.5
Dextrose	g/L	*C*	1.0	1.5	2.0
NaCl	g/L	*D*	5.0	6.0	7.0
Dipotassium phosphate	g/L	*E*	0.75	1.0	1.25

**Table:** (b)

Standard order	Experimental values					Protease production (U/mL)
	*A*	*B*	*C*	*D*	*E*	
1	7	5.5	1	7	1.25	1164.83 ± 0.21
2	3	5.5	2	5	1.25	1018.50 ± 0.13
3	7	4.5	2	7	0.75	1305.50 ± 0.17
4	3	5.5	1	7	1.25	1129.17 ± 0.09
5	3	4.5	2	5	1.25	878.00 ± 0.25
6	3	4.5	1	7	0.75	1065.83 ± 0.32
7	7	4.5	1	5	1.25	709.50 ± 0.08
8	7	5.5	1	5	0.75	932.50 ± 0.02
9	7	5.5	2	5	0.75	1134.50 ± 0.22
10	3	5.5	2	7	0.75	1409.50 ± 0.17
11	7	4.5	2	7	1.25	1231.17 ± 0.13
12	3	4.5	1	5	0.75	748.167 ± 0.07

**Table:** (a)

Variable	Experimental ranges and levels				
	−2.378	−1	0	+1	+2.378
Tryptone	0.24	3.00	5.00	7.00	9.76
Soya peptone	3.81	4.50	5.00	5.50	6.19
Dextrose	0.31	1.00	1.50	2.00	2.69
NaCl	3.62	5.00	6.00	7.00	8.38
Dipotassium phosphate	0.41	0.75	1.00	1.25	1.59

**Table:** (b)

Std. order	Experimental coded values of different variables					Protease activity (U/mL)	
	*A*	*B*	*C*	*D*	*E*	Actual	Predicted
1	−1	−1	−1	−1	−1	1198.0 ± 0.03	1187.04
2	1	−1	−1	−1	−1	1336.7 ± 0.02	1327.02
3	−1	1	−1	−1	−1	1338.5 ± 0.04	1329.21
4	1	1	−1	−1	−1	1232.0 ± 0.11	1221.26
5	−1	−1	1	−1	−1	1108.0 ± 0.15	1128.77
6	1	−1	1	−1	−1	1355.0 ± 0.09	1390.44
7	−1	1	1	−1	−1	1153.0 ± 0.15	1167.55
8	1	1	1	−1	−1	1185.0 ± 0.21	1181.28
9	−1	−1	−1	1	−1	1284.2 ± 0.06	1321.81
10	1	−1	−1	1	−1	1425.0 ± 0.24	1432.92
11	−1	1	−1	1	−1	1437.0 ± 0.02	1488.54
12	1	1	−1	1	−1	1364.0 ± 0.12	1351.72
13	−1	−1	1	1	−1	1178.0 ± 0.31	1152.72
14	1	−1	1	1	−1	1398.0 ± 0.06	1385.53
15	−1	1	1	1	−1	1234.0 ± 0.17	1216.07
16	1	1	1	1	−1	1189.0 ± 0.24	1200.94
17	−1	−1	−1	−1	1	1171.0 ± 0.32	1173.58
18	1	−1	−1	−1	1	1223.0 ± 0.09	1272.75
19	−1	1	−1	−1	1	1322.0 ± 0.03	1342.36
20	1	1	−1	−1	1	1186.0 ± 0.27	1193.59
21	−1	−1	1	−1	1	1222.0 ± 0.21	1260.05
22	1	−1	1	−1	1	1568.0 ± 0.15	1480.9
23	−1	1	1	−1	1	1345.0 ± 0.21	1325.44
24	1	1	1	−1	1	1278.0 ± 0.24	1298.36
25	−1	−1	−1	1	1	1202.0 ± 0.26	1178.03
26	1	−1	−1	1	1	1245.0 ± 0.19	1248.34
27	−1	1	−1	1	1	1365.0 ± 0.15	1371.38
28	1	1	−1	1	1	1210.0 ± 0.22	1193.75
29	−1	−1	1	1	1	1119.0 ± 0.25	1153.68
30	1	−1	1	1	1	1314.0 ± 0.09	1345.68
31	−1	1	1	1	1	1235.5 ± 0.08	1243.64
32	1	1	1	1	1	1185.0 ± 0.04	1187.7
33	−2.378	0	0	0	0	1325.0 ± 0.16	1289.12
34	2.378	0	0	0	0	1379.0 ± 0.12	1389.05
35	0	−2.378	0	0	0	1289.0 ± 0.15	1267.98
36	0	2.378	0	0	0	1254.0 ± 0.27	1249.18
37	0	0	−2.378	0	0	1357.0 ± 0.21	1335.33
38	0	0	2.378	0	0	1263.0 ± 0.17	1258.84
39	0	0	0	−2.378	0	1145.0 ± 0.28	1138.25
40	0	0	0	2.378	0	1186.0 ± 0.13	1166.92
41	0	0	0	0	−2.378	1152.0 ± 0.22	1141.46
42	0	0	0	0	2.378	1125.0 ± 0.27	1109.71
43	0	0	0	0	0	1498.0 ± 0.19	1569.75
44	0	0	0	0	0	1596.0 ± 0.15	1569.75
45	0	0	0	0	0	1578.0 ± 0.14	1569.75
46	0	0	0	0	0	1586.0 ± 0.15	1569.75
47	0	0	0	0	0	1579.0 ± 0.15	1569.75
48	0	0	0	0	0	1584.0 ± 0.16	1569.75
49	0	0	0	0	0	1574.0 ± 0.16	1569.75
50	0	0	0	0	0	1580.0 ± 0.16	1569.75

**Table 3:** Estimated effect, contribution, and ANOVA for Plackett-Burman design experiment for the five chemical components.

Source	% Contribution	Studentized effect	Ranking	Sum of squares	Mean squares	df	*F* value	*P* value prob. > *F*	Coefficient estimate	
Model				5.03*E* + 05	1.01*E* + 05	5	19.00	0.0013		Significant
*A*-Tryptone	0.87	38.14	4	4720.33	4720.33	1	0.89	0.3817	19.83	
*B*-Soya peptone	11.96	141.81	3	60208.33	60208.33	1	11.36	0.0150	70.83	
*C*-Dextrose	24.89	204.53	2	1.25*E* + 05	1.25*E* + 05	1	23.57	0.0028	102.00	
*D*-NaCl	58.71	314.14	1	2.95*E* + 05	2.95*E* + 05	1	55.71	0.0003	156.83	
*E*-Dipotassium phosphate	3.57	−77.47	5	18408.33	18408.33	1	3.47	0.1116	−39.17	
Residual				31787.67	5297.94	6				

Corrected total				5.35*E* + 05		11				

**Table 4:** Regression analysis for the production of alkaline metalloprotease from *Xenorhabdus  indica* KB-3 for quadratic response surface model fitting (ANOVA).

Source	Sum of squares	df	Mean squares	*F* value	*P* value prob. > *F*	
Model	9.952*E* + 005	17	58540.22	72.44	<0.0001	Significant
*A*-Tryptone	16990.30	1	16990.30	21.03	<0.0001	
*B*-Soya peptone	179.38	1	179.38	0.22	0.6407	
*C*-Dextrose	14027.31	1	14027.31	17.36	0.0002	
*D*-NaCl	2732.31	1	2732.31	3.38	0.0752	
*E*-Dipotassium phosphate	3196.94	1	3196.94	3.96	0.0553	
*AB*	1.129*E* + 005	1	1.129*E* + 005	139.68	<0.0001	
*AC*	24780.95	1	24780.95	30.67	<0.0001	
*AE*	5240.32	1	5240.32	6.48	0.0159	
*BC*	17302.65	1	17302.65	21.41	<0.0001	
*CD*	20175.38	1	20175.38	24.97	<0.0001	
*CE*	36106.56	1	36106.56	44.68	<0.0001	
*DE*	28770.01	1	28770.01	35.60	<0.0001	
*A* ^2^	98473.27	1	98473.27	121.86	<0.0001	
*B* ^2^	1.692*E* + 005	1	1.692*E* + 005	209.37	<0.0001	
*C* ^2^	1.300*E* + 005	1	1.300*E* + 005	160.90	<0.0001	
*D* ^2^	3.036*E* + 005	1	3.036*E* + 005	375.71	<0.0001	
*E* ^2^	3.441*E* + 005	1	3.441*E* + 005	425.80	<0.0001	
Residual	25858.54	32	808.08			
Lack of fit	19313.66	25	772.55	0.83	0.6663	Not significant
Pure error	6544.87	7	934.98			

Corrected total	1.021*E* + 006	49				

*AB*, *AC*, *AD*, *AE*, *BC*, *BD*, *BE*, *CD*, *CE*, and *DE* represent the interaction effect of variables *A*, *B*, *C*, *D*, and *E*; *A* ^2^, *B* ^2^, *C* ^2^, *D* ^2^, and *E* ^2^ are the square effects of the variables. *R ^2^ = 0.9747; adj. *R* ^2^ = 0.9612; CV = 2.17%; adequate precision ratio = 27.296; SD = 28.43; mean = 1311.20.
